# Piecing Together the Potassium Puzzle: The Weak Association Between Dietary Potassium and Hyperkalemia

**DOI:** 10.1016/j.ekir.2023.01.028

**Published:** 2023-02-02

**Authors:** Shivam Joshi, John Sebastian Babich, Jenny Shen, Kamyar Kalantar-Zadeh

**Affiliations:** 1 Department of Veterans Affairs, Orlando, Florida, USA; 2 Department of Medicine, New York University Grossman School of Medicine, New York, New York, USA; 3 SUNY Upstate Medical University Norton College of Medicine, Syracuse, New York, USA; 4 Division of Nephrology and Hypertension, The Lundquist Institute at Harbor-UCLA Medical Center, Torrance, California, USA

See Clinical Research on Page 584

Restriction of dietary potassium in kidney disease is a hotly debated topic. Historically, restriction of dietary potassium had been universal until recently. Emerging evidence over the past decade suggests that dietary potassium may not play as large a role in causing hyperkalemia as previously assumed.^1^ To that end, there has been emerging interest in exploring the potentially salutary role of plant-based diets, including the plant-dominant low-protein diet (abbreviated “PLADO”), for nutritional management of chronic kidney disease (CKD), as opposed to traditional low-potassium renal diets that have less plant diets to prevent potassium load.^2^

In this issue, Ogata *et al.*^3^ have added to this growing knowledge base by publishing their findings on the association of 24-hour urinary potassium, a proxy for daily dietary potassium, and serum potassium in patients with kidney disease. In this retrospective cohort study of Japanese patients with CKD stages 3 to 5, 24-hour measurements of urinary potassium were done 3 or 7 times with a median follow-up period of 3.9 or 8.9 months, respectively. Serum potassium levels were measured after completing each 24-hour urine collection. In total, Ogata *et al.*^3^ were able to include data from 290 participants (870 urine collections) for 3-time measurements and 220 participants (1540 urine collections) for 7-time measurements.

After adjustment for several variables, including comorbidities like diabetes and hypertension and the use of medications that could affect potassium levels, the authors show a mean increase in serum potassium of 0.12 mEq/l (for 3-time measurements) for every 10 mEq (0.4 grams) per day increase in continuous 24-hour urinary potassium excretion. Further, they showed that this risk increased with worsening CKD stage as follows: 0.08, 0.12, and 0.16 mEq/l for every 10 mEq/d increase in continuous 24-hour urinary potassium excretion for CKD stages 3, 4, and 5, respectively. Similar findings were seen for 7-time measurements. However, the associations were relatively weak with unadjusted R^2^ of 0.08, 0.14, and 0.18 for CKD stages 3, 4, and 5, respectively.

The information provided by Ogata *et al.*^3^ fills an important void in the literature, notably, the association of dietary potassium, as measured by multiple 24-hour urine collections, with serum potassium levels in persons with CKD. The study overcomes the issues of other studies that rely on single urinary or serum measurements. Notwithstanding clinically relevant data presented in this important research, the study is limited by a lack of corresponding dietary information including plant versus animal protein, reliance on a single-center, and a lack of information on other variables that could potentially affect serum potassium levels, including the presence or absence of metabolic acidosis, constipation, and hyperglycemia. For example, metabolic acidosis, a common complication of advanced CKD, is a strong mediator of more severe hyperkalemia and could potentially explain some of the associations seen.^4^ Although the authors adjust for the usage of bicarbonate, no information is provided regarding the therapeutic effect. Further, it is somewhat unusual to see only 2 of a total of 510 patients with CKD using oral bicarbonate.

The information provided by Ogata *et al.*^3^ largely fits within existing knowledge of dietary potassium and its association with serum potassium. Noori *et al.*^5^ also found a weak association (*r* = 0.14) with dietary potassium and serum potassium in a secondary analysis of 224 patients on dialysis. However, beyond this, additional cases of hyperkalemia are often limited to case reports or isolated instances with additional factors, like potassium-raising medications, at play.^1^

It is important to note that Ogata *et al.*^3^ accounted for the usage of potassium-containing supplements, which behave different than food. Potassium-containing supplements are thought to have higher bioavailability of potassium (approaching 100%) compared to standard omnivorous diets, which are estimated to have potassium bioavailability of approximately 77%.^6^ However, the study by Ogata *et al.*^3^ does not provide any information on the extent of potassium added as additives to foods. Potassium added to foods would be reflected in a 24-hour urine collection; it would help clarify which foods to restrict for patients with kidney disease. For example, unprocessed plant foods, which have often been restricted in traditional renal diets have a lower potassium bioavailability of approximately 65%.^1^ Indeed, previously published information from the International Study of Macro/Micronutrients and Blood Pressure (INTERMAP) has shown that the largest source of dietary potassium in the Japanese diet was from fruits and vegetables, accounting for 45% of all dietary potassium consumed, in participants without kidney disease.^7^ However, it is unclear if this same dietary pattern occurred in the cohort studied by Ogata *et al.*^3^ because dietary potassium intake appears to have been lower than what was seen in INTERMAP. Although the authors attempted to control for reverse causation in their model, it is possible that the effects of reverse causation could still have played a role.

Ogata *et al.*^3^ are wise to suggest that their “findings are insufficient to support or oppose any dietary approaches to hyperkalemia.” However, their study adds to a growing body of evidence that suggests a weak association of dietary potassium with serum potassium. Further research is needed to answer ongoing questions regarding the applicability of potassium-rich diets in patients with kidney disease.

## Disclosure

SJ has received honorarium for consulting from Insyght Interactive, Otsuka, AstraZeneca, Vifor Pharma, and Med Learning Group. The contents of this article do not represent the views of the Department of Veterans Affairs or the United States Government. KKZ has received honoraria and/or support from Abbott, Abbvie, ACI Clinical Cara Therapeutics, Agency of Healthcare Quality and Research (AHRQ), Akebia, Alexion, Amgen, Ardelyx, American Society of Nephrology, Astra-Zeneca, Aveo, BBraun, Chugai, Cytokinetics, Daiichi, DaVita, Fresenius, Genentech, GSK, Haymarket Media, Hofstra Medical School, International Federation of Kidney Foundations, International Society of Hemodialysis, International Society of Renal Nutrition & Metabolism, Japanese Society of Dialysis Therapy, Hospira, Kabi, Keryx, Kissei, Novartis, Novo-Nordisk, OPKO, National Institutes of Health, NKF (National Kidney Foundations), Pfizer, Regulus, Relypsa, Resverlogix, Dr Schaer, Sandoz, Sanofi, Shire, Veterans’ Affairs, Takeda, Vifor, UpToDate, ZS-Pharma. JSB declared no competing interests. JIS has received honoraria from Healthmap Solutions, outside the submitted work.
